# Parentage Reconstruction in *Eucalyptus nitens* Using SNPs and Microsatellite Markers: A Comparative Analysis of Marker Data Power and Robustness

**DOI:** 10.1371/journal.pone.0130601

**Published:** 2015-07-09

**Authors:** Emily J. Telfer, Grahame T. Stovold, Yongjun Li, Orzenil B. Silva-Junior, Dario G. Grattapaglia, Heidi S. Dungey

**Affiliations:** 1 Scion (New Zealand Forest Research Institute Ltd.), Whakarewarewa, Rotorua, New Zealand; 2 Laboratório de Genética Vegetal, EMBRAPA Recursos Genéticos e Biotecnologia, Brasília, Brazil; 3 Programa de Ciências Genômicas e Biotecnologia, Universidade Católica de Brasília, Brasilia, Brazil; USDA/ARS, UNITED STATES

## Abstract

Pedigree reconstruction using molecular markers enables efficient management of inbreeding in open-pollinated breeding strategies, replacing expensive and time-consuming controlled pollination. This is particularly useful in preferentially outcrossed, insect pollinated *Eucalypts* known to suffer considerable inbreeding depression from related matings. A single nucleotide polymorphism (SNP) marker panel consisting of 106 markers was selected for pedigree reconstruction from the recently developed high-density *Eucalyptus* Infinium SNP chip (EuCHIP60K). The performance of this SNP panel for pedigree reconstruction in open-pollinated progenies of two *Eucalyptus nitens* seed orchards was compared with that of two microsatellite panels with 13 and 16 markers respectively. The SNP marker panel out-performed one of the microsatellite panels in the resolution power to reconstruct pedigrees and out-performed both panels with respect to data quality. Parentage of all but one offspring in each clonal seed orchard was correctly matched to the expected seed parent using the SNP marker panel, whereas parentage assignment to less than a third of the expected seed parents were supported using the 13-microsatellite panel. The 16-microsatellite panel supported all but one of the recorded seed parents, one better than the SNP panel, although there was still a considerable level of missing and inconsistent data. SNP marker data was considerably superior to microsatellite data in accuracy, reproducibility and robustness. Although microsatellites and SNPs data provide equivalent resolution for pedigree reconstruction, microsatellite analysis requires more time and experience to deal with the uncertainties of allele calling and faces challenges for data transferability across labs and over time. While microsatellite analysis will continue to be useful for some breeding tasks due to the high information content, existing infrastructure and low operating costs, the multi-species SNP resource available with the EuCHIP60k, opens a whole new array of opportunities for high-throughput, genome-wide or targeted genotyping in species of *Eucalyptus*.

## Introduction

*Eucalyptus nitens* (Dean et Maiden) Maiden has shown promise as a vigorous species for planted forests, well-suited to many New Zealand sites. Early introductions of this species to New Zealand showed spectacular early growth, good form characteristics and cold hardiness. Performance of this species in Australia indicated promise for both pulp and sawn-timber production. Provenance testing in NZ began in 1979 [1]. Early results from these trials indicated that provenances from Central Victoria would be most suited to New Zealand conditions. New South Wales provenances potentially had better growth rates, but were more severely attacked by *Paropsis charybdis* Stål (Coleoptera: Chrysomelidae: Paropsini). Until the 1990’s there was little confidence in planting *E*. *nitens* because of periodic severe defoliation by *P*. *charybdis* (hereafter referred to as *Paropsis*). In the 1990s a biological control agent was successfully introduced, and interest in the species was re-kindled. A second round of progeny testing was completed and the best material grafted into seed orchards. The biological control was itself overcome by a hyperparasite, and currently the growing of *E*. *nitens* is only carried out in cooler areas where *Paropsis* does not thrive [2].

*Eucalyptus nitens* is insect-pollinated across an estimated mean distance of 42 m [3,4]; in most instances, this limits the genetic pool to within a single seed orchard. *Eucalypts* open-pollinated progenies are known to include up to 20–30% selfed individuals which can suffer from considerable inbreeding depression, both early and late-acting [5,6,7]. Therefore, heritability estimates commonly include a correction for inbreeding [8]. Around 2004 the availability of seed from seed orchards and clonal archives prompted a review of the breeding strategy for *E*. *nitens* (Stovold, et al., unpublished breeding plan). It was decided to establish the next generation of progeny tests using seed collected from orchards and archives, rather than pursue an expensive and difficult controlled crossing programme, and to split the population into two smaller trials, separated in time to spread costs (a rolling front approach [9]). A key component of this approach was based on using forward selection coupled to DNA marker genotyping to control inbreeding amongst the selections and hence among the next generation. The concept was dependent on a previously successful study using 13 microsatellite markers to identify male parentage in seed orchard seed, which would be used as a method to control inbreeding and maintain genetic diversity in the next generation of selections [1].

Microsatellite markers have been the major tool for a number of genetic analyses, including verification of clonal identity, pedigree reconstruction, monitoring genetic diversity, inbreeding, population structure, and detection and quantification of linkage disequilibrium [10]. This was due to their high multiallelism that translates in high polymorphic information content (PIC) [11] and heterozygosity. Recent advances in ‘next-generation’ sequencing technologies have greatly reduced the cost associated with identifying polymorphic loci, including microsatellites, insertion/deletions (INDELS) and single nucleotide polymorphisms (SNPs). In particular, SNP markers are plentiful and are also widely dispersed throughout the genome, although being principally biallelic, they individually yield limited polymorphic information [12]. However, the increasing ease, reliability and speed with which they can be multiplexed and then genotyped across large numbers of samples is currently driving a switch towards genetic analyses performed with many SNP markers rather than using more laborious microsatellite marker sets [10].

Recent studies have compared the performance of microsatellites and SNPs. In bread wheat, analyses of population structure and genetic diversity performed using both microsatellites and SNPs gave different results and analysis of linkage disequilibrium required greater numbers of SNPs to ensure adequate chromosomal coverage [13]. Analysis of genetic diversity and population structure in wine grape was performed with both microsatellites and SNPs, which were both able to distinguish two subspecies, although the estimates of heterozygosity differed between the two marker types [14]. In both maize [15] and citrus fruit trees [16], genetic relatedness using both marker types was compared. In general, microsatellites by virtue of their higher PIC [11] and heterozygosity, performed better. However, similar results for population structure could be achieved by increasing the number of SNPs to approximately 10 times the number of microsatellites [17,18]. SNPs out-performed microsatellites in data reproducibility, as well as in cost-effectiveness and flexibility of scale. [15,16]. Increasing numbers of commercially available technologies with good transferability of markers between platforms are providing cost-effective options for SNP genotyping [19]. These technologies provide real competition in the market as well as ability to scale from tens of samples and tens of SNP markers, up to thousands of samples and millions of SNPs.

*Eucalyptus* genomics has now entered the post-genome era [20]. Estimates of SNP abundance in *Eucalypts* are extremely high; as high as one SNP every 16 base pairs (bp) in one study [21], and one SNP every 45 bp in another [22] depending on the species and the extent of intra-specific diversity sampled. The abundance of markers with which to develop vast genotyping panels has also benefited by the high degree of transferability observed for SNPs in *Eucalypts* [23,24]. The application of SNP markers builds on a rich history of molecular marker application in *Eucalypts*; from early application of microsatellites for parentage reconstruction in *E*. *grandis* [25,26,27], Diversity Array Technology (DArT) markers to examine diversity [24,28], and finally arriving at SNP markers which are being applied in wider and wider numbers as development and genotyping costs continue to decrease [29].

Initially, small numbers of SNP markers were examined in candidate genes, such as in Külheim et al. [21] study into secondary metabolites. Larger numbers of SNPs have been detected from a range of traditional Sanger and next generation sequencing (NGS) resources and examined using Illumina’s Golden-Gate genotyping technology [23] and now a large *Eucalyptus* SNP chip platform (EuCHIP60K) that allows high-throughput, genome-wide SNP genotyping in 14 different eucalypt species using Illumina’s Infinium technology [30]. Application of SNPs in *Eucalyptus* breeding programs could include precise parentage assignment and reconstruction of pedigrees. Correia et al. [31] showed that a panel of 35 SNPs would provide a probability of parentage exclusion >99% in *Eucalyptus grandis*, matching the performance of commonly used 17-microsatellite marker sets, while Thavamanikumar et al. [22] suggested that 20 SNPs could be sufficient. Besides pedigree reconstruction, the most significant use of genome-wide SNP genotyping technology in breeding will be the operational implementation of genomic selection [32,33].

In this study we examined the efficacy of the two commonly used DNA markers, SNPs and microsatellites, for parentage reconstruction in a highly heterozygous forest tree species, *Eucalyptus nitens*. While a few studies have looked at this topic in wild animals [34,35], no studies to date have specifically examined the comparative performance of these widely used sequence polymorphism assays in plants. We compared two different microsatellite marker sets, one developed by Gea et al. (2007) [1] and a second one involving a combination of EMBRA markers specifically selected for routine genotyping from the initial microsatellite developments [36] with a set of SNPs selected from the high-density *Eucalyptus* SNP chip EuCHIP60K [30]. We utilised offspring from two New Zealand seed orchards to assess the efficiency with which pollen parents could be identified with the two kinds of molecular markers. We examined the relative contribution of different pollen parents in the progeny tested and discuss the results in the context of the New Zealand *E*. *nitens* breeding program.

## Materials and Methods

### Trial design and selection

All *Eucalyptus nitens* samples were collected from privately owned seed orchards or progeny trials planted on private land. The Tinkers and Fortification Rd samples were owned by Southwood Export Ltd. and owner Graham Manley was involved in sample collection. The Alexandra samples were collected from Conroys Rd. Nursery, with the permission and support of owner Mike Olsen. The progeny collected from the Scion nursery were our own samples.

### Seed orchards

Open-pollinated seeds were collected in 2004 from two clonal seed orchards: Tinkers (46°16.1’ S 169°1.6’ E), and Alexandra (45°15.6’ S 169°24.3’ E). Both orchards were established with grafted ramets from the previous generation of trials.

### Progeny selected

Open-pollinated seedlings were raised at the Scion nursery in 2005 (38°9.5’ S 176°16.1’ E) and a trial was established at Fortification in November 2005 (46°30.5 S 168°59.9 E). In February 2011 this trial was assessed for diameter, straightness, and stem malformation and parental breeding values were estimated for all traits (Baltunis, unpublished data). Selections for the next generation were made based on selection of the best tree in each family. Leaves were collected for downstream DNA analysis. As discussed in Gea, *et al*. [1], *E*. *nitens* is insect-pollinated across an estimated mean distance of 42 m [3,4]. Therefore, provided all the parental genotypes within an orchard are sampled and a buffer zone is adopted to prevent pollen flow from neighbouring orchards, all possible parents should be captured.

### Genomic DNA extraction

From the Alexandra orchard, 9 open-pollinated (OP) offspring and 18 putative parents were sampled. From the Tinkers orchard, 17 OP offspring and 29 putative parents were sampled. The seed parents of these offspring were recorded but without 100% confirmation. The putative parents included pollen parents and the recorded seed parents. Genomic DNA (gDNA) from the parental trees was extracted from leaf tissue using a CTAB (cetyltrimethyltetraammonium bromide) buffer as described by Gea et al. [1]. For the *E*. *nitens* offspring, gDNA was extracted using a high-throughput method developed from the commercially available NucleoSpin Plant II kit (Machery-Nagel, Düren, GER) [37].

### Genotyping

#### Microsatellite marker genotyping

The Scion *Eucalyptus nitens* microsatellite markers were developed into a 13-marker multiplex panel and genotyped as described in Gea et al. [1]. Prior to 2007, individuals were genotyped using ABI PRISM 3100 Genetic Analyzer (Life Technologies, Carlsbad, California, U.S.) and the following Life Technologies consumables: POP-4 Polymer, GeneScan 500 LIZ size standard. Alleles were detected visually using GeneScan v3.7 and Genotyper v3.7 software (Life Technologies). Alleles for the 13 microsatellite markers genotyped after 2007 were separated and detected on an ABI PRISM 3130*xl* Genetic Analyzer using the following Life Technologies consumables: POP-7 Polymer, GeneScan 600 LIZ size standard. Alleles were detected automatically using the GeneMapper v4.1 software package (Life Technologies). Only microsatellite genotypes obtained post-2007 have been used for the pedigree reconstruction analysis; however, we have compared the pre- and post-2007 genotypes of the 13 microsatellite markers to highlight how changes in technology have affected allele calling over time.

A set of 16 *Eucalyptus* EMBRA microsatellites developed by Brondani et al. [36] was specifically selected and optimized into a 3 multiplex panels and genotyped at the EMBRAPA lab as described in [38]. Alleles for the 16 microsatellites genotyped were separated and detected on an ABI PRISM 3100*xl* Genetic Analyzer using POP-6 Polymer, using a custom-made ROX-labeled size standard [39] and data collected under dye set D spectral calibration using GeneScan v3.7 and analyzed with Genotyper v3.7 (Life Technologies).

#### SNP marker genotyping

Genomic DNA samples (500ng) from 47 putative parents, including two pairs of replicate samples, and 26 progeny from two seed orchards were genotyped by GeneSeek, Inc. (a Neogene company, Lincoln, NE, USA) using the Illumina Infinium EuCHIP60K *Eucalyptus* SNP chip [30]. Call rates for individual samples, call rates for individual SNPs, mistyping between duplicate samples, average GenCall scores and allele frequency per SNP were calculated. GenCall (GC) scores are the quality metric generated for each individual genotype, based on the quality of the clustering in Illumina’s [40] GenomeStudio data analysis software. GC scores range from 0 to 1, with scores of less than 0.15 being discarded as failed assays.

### Marker selection for parentage reconstruction

#### Microsatellite selection

Following genotyping, 9 and 10 markers from the Scion microsatellite marker set with call rate greater than 70% were used for parentage reconstruction analysis in the Alexandra and Tinkers seed orchards respectively. For the EMBRA microsatellite marker set, 14 markers with call rates greater than 70% were used for parentage reconstruction analysis in both seed orchards.

#### SNP selection

A set of 106 SNP markers was selected from the EuCHIP60K *Eucalyptus* SNP chip genotypic dataset for parentage reconstruction. The criteria for selecting SNPs were as follows: the observed minimum allele frequency (MAF) between 0.45 and 0.55, GC score average > 0.85, and sample call rate >0.95. In addition, selected SNPs had to adhere to Hardy-Weinberg equilibrium (HWE) as assessed using a Chi-square goodness-of-fit test, which tested the significance of the differences between the observed and expected frequencies [41]. SNP markers were included if the *p*-value of this test was larger than 0.10. Finally, selected SNP markers had to show no evidence of linkage assessed by a squared correlation of allele frequencies r^2^< 0.11 used to measure linkage disequilibrium [42].

### Parentage analysis using exclusion probability

We used exclusion probability analysis to determine the most likely two parents of each progeny individual using both microsatellite markers panels and the select set of SNP markers. Expected heterozygosity (*H*_*exp*_), polymorphism information content (*PIC*) and exclusion probability (*EP*) were calculated for SNP and microsatellite markers. Expected heterozygosity was calculated as $H_{exp}\;=\;1-\sum p_{ij}^{2}$ [43], where *p*_*ij*_ is the observed frequency of allele *i* on locus *j*, and *PIC* was estimated as in Botstein et al. [11]

$$PIC\;=\;H_{exp}-\sum_{i\;=\;1}^{n-1}p_{i}^{2}\sum_{j\;=\;i+1}^{n}p_{j}^{2}$$

where *p*_*i*_ and *p*_*j*_ are observed allelic frequencies of locus *i* and *j*, *n* is the number of loci.

Exclusion probability is the probability of excluding a random individual from the population as a potential parent of an individual based on the genotypes of two parents and one offspring. The probability of excluding a parent given two parents and one offspring (*EP*_*A*_) was calculated as in [44]:

$${EP}_{A}\;=\;1-2\sum_{i\;=\;1}^{n}p_{i}^{2}+\sum_{i\;=\;1}^{n}p_{i}^{3}+\sum_{i\;=\;1}^{n}p_{i}^{4}-3\sum_{i\;=\;1}^{n}p_{i}^{5}-2(\sum_{i\;=\;1}^{n}p_{i}^{2}){\;}^{2}+3\sum_{i\;=\;1}^{n}p_{i}^{2}\sum_{i\;=\;1}^{n}p_{i}^{3}$$

The probability of excluding both parents given a trio of two parents and one offspring (${EP}_{B}$) was calculated as in Jamieson and C S Taylor [45]:

$${EP}_{B}\;=\;1+4\sum_{i\;=\;1}^{n}p_{i}^{4}-4\sum_{i\;=\;1}^{n}p_{i}^{5}-3\sum_{i\;=\;1}^{n}p_{i}^{6}-8(\sum_{i\;=\;1}^{n}p_{i}^{2}){\;}^{2}+8\sum_{i\;=\;1}^{n}p_{i}^{2}\sum_{i\;=\;1}^{n}p_{i}^{3}+2(\sum_{i\;=\;1}^{n}p_{i}^{3}){\;}^{2}$$

The probability of excluding a parent and offspring relationship when one parental genotype is unavailable (*EP*_*C*_) was calculated as in Jamieson and C S Taylor [45]:

$${EP}_{C}\;=\;1-4\sum_{i\;=\;1}^{n}p_{i}^{2}+2(\sum_{i\;=\;1}^{n}p_{i}^{2}){\;}^{2}+4\sum_{i\;=\;1}^{n}p_{i}^{3}-3\sum_{i\;=\;1}^{n}p_{i}^{4}$$

Combining exclusion probability (*P*) over *k* unlinked markers in any of above formulae gives:

$$P\;=\;1-(1-{EP}_{1})(1-EP_{2})\;(1-{EP}_{3})∙∙∙∙(1-EP_{k})$$

The genotypes of the individual progeny were compared with the genotypes of 18 putative parents in Alexandra and 29 putative parents in Tinkers. Most likely parent or most likely trio was the one with the least number of exclusions or inconsistent loci. Maternal parentage was deemed to be supported if the recorded seed parent was identified as one of the two most likely parents for an individual. The likelihood of the pollen parent being correctly identified was supported by consistency between the recorded seed parent and the most likely seed parent based on exclusion analysis.

## Results

### Data quality

#### SNP chip

Despite the use of both CTAB and commercially available kits to extract DNA from parents and offspring respectively, the genotypic call rate using the EuCHIP60K *Eucalyptus* SNP chip showed remarkably little variation between samples. The average call rate was 84.62% with a standard deviation of 0.0027. The EuCHIP60K was designed as a multi-species assay [30], and we would not expect all SNP probes on the chip to be assayable in *E*. *nitens*. To test the reproducibility of the SNP marker genotypes, two pairs of replicate DNA samples were genotyped. We observed four discrepancies out of the 54,832 genotyped SNPs for the replicates of parental tree 897.164 which equates to a very low mistyping rate of 0.0073%. For the replicate samples of parental tree 896.829, we also observed four discrepancies out of 54,149 genotyped SNPs which equates to a mistyping rate of 0.0074%. In addition, the GC scores for all four of the mismatched SNPs were below 0.85 in both cases. However when we only examined the 106 SNPs selected for the pedigree reconstruction analysis the mistyping rate dropped to 0%.

#### Scion microsatellite

For the 13 microsatellite markers genotyped pre-2007, the fail rate across all genotypes was 5% (72 missing data points out of 1372 alleles genotyped), the post-2007 genotypic dataset had a fail rate of 25% (786 missing data points out of 3108 alleles genotyped). Across all samples with a genotype generated in both the pre-2007 and post-2007 microsatellite datasets, the mistyping rate was 29% and included missing data points, shifts in allele size and loss of second allele in a number of previously heterozygous loci. An example from a single genotype showing changes in allele calls over time is given in S1 Table. Even within the dataset generated post-2007, the mistyping rate between replicates was 10% or 3/28 alleles, including both missing data points and loss of second allele in a number of previously heterozygous loci (S2 Table).

#### EMBRA microsatellite

For the 16 microsatellite markers genotyped at EMBRAPA, the fail rate across all genotypes was 10% (320 missing data points out of a total 3072 alleles genotyped). Replicate samples genotyped at EMBRAPA had a mistyping rate of 3% or 1/32 alleles genotyped (S3 Table). A single marker in common between the two microsatellite marker panels was EMBRA10, the primer sequences for which are identical except for a 7 base PIG tail added to the Scion reverse primer [1,46]. Adjusted for the 7 base differences in size, we compared a set of samples genotyped with EMBRA10 at both Scion post-2007 and EMBRAPA and found a mistyping rate of 74%, however this included a large number of failed results in the EMBRA10 results from EMBRA (call rate of only 36%). When the genotypes of EMBRA10 were compared in individuals with allele calls from the both laboratories, the mistyping rate was 32% (16/50 alleles genotyped) (S4 Table)

### Polymorphisms of SNPs and microsatellites

Expected heterozygosity, polymorphic information content and combined exclusion probabilities of SNPs and microsatellites in Alexandra and Tinkers are listed in Table 1. Within the Scion microsatellite panel, the number of alleles per microsatellite marker ranged from 2 to 6 with an average of 4.33 for Alexandra and from 3 to 16 with an average of 6.79 for Tinkers. Within the EMBRA microsatellite panel, the number of alleles per microsatellite marker ranged from 2 to 16 with an average of 9.79 for Alexandra and from 3 to 17 with an average of 10.50 for Tinkers. As expected, both the observed and expected heterozygosities were higher in both microsatellite panels than SNPs, but lower combined exclusion probabilities were observed in Scion microsatellites than EMBRA microsatellites and SNPs. The lower exclusion probabilities in the Scion microsatellites set when compared to the EMBRA microsatellites and SNPs reflects the lower number of alleles and might also be due to the comparatively higher rate of missing genotypes (Table 2).

**Table 1:** Summary of the SNP and Microsatellite marker metrics. Number of individuals genotyped, number of markers, mean number of loci, mean expected heterozygosity (*H*_*exp*_), mean polymorphic information content (*PIC*), combined probabilities of excluding one parent (*EP*_*A*_) and excluding both parents (*EP*_*B*_) given a trio of genotypes of two parents and an individual offspring, combined probability excluding parent and offspring relationship given one putative parent and one offspring (*EP*_*C*_) for the SNPs and microsatellites.

	SNPs		Scion Microsatellites		EMBRA Microsatellites	
	Alexandra	Tinkers	Alexandra	Tinkers	Alexandra	Tinkers
**No of individuals**	27	45	27	45	27	48
**No of markers**	106	106	9	10	14	14
**Mean No of alleles per locus**	2	2	4.33 (2–6)	6.79 (3–16)	9.79 (2–16)	10.50 (3–17)
**No of markers deviating from HWE**	0	0	1	4	14	14
**Mean *H***_***exp***_	0.493	0.499	0.584	0.650	0.737	0.727
**Mean *PIC***	0.367	0.372	0.530	0.606	0.712	0.700
**Combined *EP***_***A***_	1.000	1.000	0.890	0.994	1.000	1.000
**Combined *EP***_***B***_	1.000	1.000	0.986	0.999	1.000	1.000
**Combined *EP***_***C***_	1.000	1.000	0.999	0.999	1.000	1.000

**Table 2:** Comparison of each Genotyping experiment.

*Genotyping Experiment*	*Confirmed seed parent*	*Missing genotypes*	*Inconsistent alleles between replicates*	*Average No. of inconsistent trios*
SNPs EuCHIP60K	N/A	15%	0.0074%	N/A
SNPs 106 panel	92%	0.03%	0%	2.8%
SCION pre 2007 microsatellites	N/A	5%	N/A	N/A
SCION post 2007 microsatellites	18%	25%	10%	26%
EMBRA microsatellites	96%	10%	3%	31%

### Pedigree reconstruction

In the Alexandra orchard, the recorded seed parent was returned as the most likely parent for 8 out of the 9 progeny individuals tested with both the 106 SNP marker dataset (Table 3) and the EMBRA microsatellite dataset (Table 4). The Scion microsatellite marker dataset only supported 3 out of 9 recorded maternal assignments (Table 5). In progeny where the recorded seed parent had been supported by the most likely parent in exclusion analysis, the average number of inconsistent SNP markers with alleles present in a progeny that could not be attributed to a parent was 5.75 (2.9%). In the case of offspring 572 and 727, all of the inconsistencies could be attributed to the putative pollen parent. Inconsistencies within the most likely trios for offspring 91 and 166 were also observed for a single SNP marker. This same SNP was also the source of the single inconsistency observed in offspring 1329 from Tinkers orchard. For the progeny with recorded seed parents supported by microsatellites, the average marker inconsistency rates between trios were 13.3% and 32% for the Scion and EMBRA panels respectively.

**Table 3:** Parentage Reconstructions using Exclusion analysis with 106 SNPs.

*Seed Orchard*	*Offspring*	*Recorded Seed Parent*	*Assigned parent 1*	*Assigned parent 2*	*Incomplete marker trios* ^1^	*Inconsistent marker trios* ^2^	*Inconsistent loci (%)* ^3^
Alexandra	91	896.804	***896*.*804***	896.815	1	1	1%
Alexandra	166	896.811	***896*.*811***	896.815	1	1	1%
Alexandra	262	896.829	***896*.*829***	896.810	0	0	0%
Alexandra	572	896.811	***896*.*811***	896.802	0	11	10%
Alexandra	727	896.810	***896*.*810***	896.800	0	12	11%
Alexandra	1543	896.829	***896*.*829***	896.811	0	0	0%
Alexandra	1870	896.807	896.800	896.829	0	21	20%
Alexandra	1969	896.800	***896*.*800***	896.815	1	0	0%
Alexandra	2049	896.815	***896*.*815***	896.827	1	0	0%
Tinkers	13	897.135	***897*.*135***	897.173	1	0	0%
Tinkers	151	897.169	***897*.*169***	897.155	0	0	0%
Tinkers	266	897.163	***897*.*163***	897.148	0	0	0%
Tinkers	547	897.164	***897*.*164***	897.148	0	0	0%
Tinkers	551	897.161	N/A	N/A	N/A	N/A	N/A
Tinkers	562	897.119	***897*.*119***	897.148	0	0	0%
Tinkers	689	897.173	***897*.*173***	897.148	0	0	0%
Tinkers	913	897.158	897.148	896.408	0	15	14%
Tinkers	923	897.153	***897*.*153***	897.144	1	0	0%
Tinkers	1082	897.101	***897*.*101***	897.144	1	0	0%
Tinkers	1105	897.110	***897*.*110***	897.144	1	0	0%
Tinkers	1283	897.144	***897*.*144***	896.423	1	0	0%
Tinkers	1288	897.109	***897*.*109***	896.408	0	0	0%
Tinkers	1329	897.164	***897*.*164***	897.156	0	1	1%
Tinkers	1471	897.134	***897*.*134***	897.173	0	0	0%
Tinkers	1548	897.145	***897*.*145***	897.163	0	0	0%
Tinkers	1910	897.141	***897*.*141***	897.150	0	0	0%
Tinkers	2090	896.423	***896*.*423***	897.173	0	15	14%

^1^ Number of individual markers where data was missing for one or more of the parents and progeny.

^2^ Number of individual markers where progeny was inconsistent with parental genotype.

^3^ Percentage of complete marker trios where progeny was inconsistent with parental genotype.

**Table 4:** Parentage Reconstruction using Exclusion analysis with 14 EMBRA microsatellites.

*Seed Orchard*	*Offspring*	*Recorded Seed Parent*	*Assigned parent 1*	*Assigned parent 2*	*Incomplete marker trios* ^1^	*Inconsistent marker trios* ^2^	*Inconsistent loci (%)* ^3^
Alexandra	91	896.804	***896*.*804***	896.815	2	3	25%
Alexandra	166	896.811	***896*.*811***	896.815	7	2	29%
Alexandra	262	896.829	***896*.*829***	896.810	4	2	20%
Alexandra	572	896.811	***896*.*811***	896.802	6	4	50%
Alexandra	727	896.810	***896*.*810***	896.829	5	5	56%
Alexandra	1543	896.829	***896*.*829***	896.811	0	3	21%
Alexandra	1870	896.807	896.802	896.810	5	7	78%
Alexandra	1969	896.800	***896*.*800***	896.815	1	3	23%
Alexandra	2049	896.815	***896*.*815***	896.827	1	5	38%
Tinkers	13	897.135	***897*.*135***	897.173	1	3	23%
Tinkers	151	897.169	***897*.*169***	897.155	2	4	33%
Tinkers	266	897.163	***897*.*163***	897.148	2	4	33%
Tinkers	547	897.164	***897*.*164***	897.148	1	3	23%
Tinkers	551	897.161	***897*.*161***	897.164	0	4	29%
Tinkers	562	897.119	***897*.*119***	897.148	2	0	0%
Tinkers	689	897.173	***897*.*173***	897.148	1	4	31%
Tinkers	913	897.158	***897*.*158***	897.148	2	3	25%
Tinkers	923	897.153	***897*.*153***	897.144	1	4	31%
Tinkers	1082	897.101	***897*.*101***	897.144	0	5	36%
Tinkers	1105	897.110	***897*.*110***	897.144	1	3	23%
Tinkers	1283	897.144	***897*.*144***	897.141	2	4	33%
Tinkers	1288	897.109	***897*.*109***	897.155	0	3	21%
Tinkers	1329	897.164	***897*.*164***	897.156	3	2	18%
Tinkers	1471	897.134	***897*.*134***	897.173	5	4	44%
Tinkers	1548	897.145	***897*.*145***	897.163	1	4	31%
Tinkers	1910	897.141	***897*.*141***	897.150	6	1	13%
Tinkers	2090	896.423	***896*.*423***	897.101	2	7	58%

^1^ Number of individual markers where data was missing for one or more of the parents and progeny.

^2^ Number of individual markers where progeny was inconsistent with parental genotype.

^3^ Percentage of complete marker trios where progeny was inconsistent with parental genotype.

**Table 5:** Parentage Reconstruction using Exclusion analysis with SCION microsatellites.

*Seed Orchard*	*Offspring*	*Recorded Seed Parent*	*Assigned parent 1*	*Assigned parent 2*	*Incomplete marker trios* ^1^	*Inconsistent marker trios* ^2^	*Inconsistent loci (%)* ^3^
Alexandra	91	896.804	***896*.*804***	896.815	6	0	0%
Alexandra	166	896.811	896.815	896.826	4	2	40%
Alexandra	262	896.829	***896*.*829***	896.810	4	0	0%
Alexandra	572	896.811	896.827	896.802	3	1	17%
Alexandra	727	896.810	896.815	896.826	4	3	60%
Alexandra	1543	896.829	896.804	896.811	5	0	0%
Alexandra	1870	896.807	896.815	896.826	4	2	40%
Alexandra	1969	896.800	896.815	896.826	4	1	20%
Alexandra	2049	896.815	***896*.*815***	896.826	4	2	40%
Tinkers	13	897.135	***897*.*135***	897.155	3	3	43%
Tinkers	151	897.169	***897*.*169***	897.155	4	0	0%
Tinkers	266	897.163	897.145	897.164	3	0	0%
Tinkers	547	897.164	897.135	897.156	4	2	33%
Tinkers	551	897.161	897.141	897.177	4	2	33%
Tinkers	562	897.119	897.145	897.164	2	2	25%
Tinkers	689	897.173	897.155	897.109	3	2	29%
Tinkers	913	897.158	897.129	897.177	4	1	17%
Tinkers	923	897.153	897.141	896.408	4	2	33%
Tinkers	1082	897.101	897.110	897.144	1	2	22%
Tinkers	1105	897.110	897.155	897.153	5	1	20%
Tinkers	1283	897.144	897.155	897.153	5	2	40%
Tinkers	1288	897.109	897.142	897.124	5	2	40%
Tinkers	1329	897.164	897.142	897.156	4	1	17%
Tinkers	1471	897.134	897.155	897.109	3	2	29%
Tinkers	1548	897.145	897.148	897.124	5	2	40%
Tinkers	1910	897.141	897.110	896.408	4	1	17%
Tinkers	2090	896.423	897.142	897.124	6	2	50%

^1^ Number of individual markers where data was missing for one or more of the parents and progeny.

^2^ Number of individual markers where progeny was inconsistent with parental genotype.

^3^ Percentage of complete marker trios where progeny was inconsistent with parental genotype.

In the Tinkers orchard, the recorded seed parent was returned as the most likely parent for 16 out of the 17 progeny tested using the 106 SNP marker dataset (Table 3). The Scion microsatellite marker dataset was only able to support 2 out of 18 recorded maternal assignments (Table 5), whilst the EMBRA microsatellite dataset outperformed the 106 SNP panel by supporting 18 out of 18 recorded seed parents as the most likely parent (Table 4). In progeny where the recorded seed parent had been supported with marker analysis, there were two instances of inconsistent SNP markers, an average of 0.9%. For the progeny with seed parents correctly identified using microsatellites, the average marker inconsistency rates between trios were 21.5% and 28% for the Scion and EMBRA panels respectively.

Overall the SNP marker set and the EMBRA microsatellites performed the best, with 24 of the progeny having the recorded seed parent supported by both of these marker sets. In 20/24 of these progeny, the same pollen parent was predicted as well (Table 6).

**Table 6:** Concurrence of parentage reconstruction between 106 SNPs and EMBRA microsatellites.

*Seed Orchard*	*Offspring*	*Recorded Seed Parent*	*106 SNPs*		*EMBRA microsatellites*	
			Assigned parent 1	Assigned parent 2	Assigned parent 1	Assigned parent 2
Alexandra	91	896.804	896.804	896.815	896.804	896.815
Alexandra	166	896.811	896.811	896.815	896.811	896.815
Alexandra	262	896.829	896.829	896.810	896.829	896.810
Alexandra	572	896.811	896.811	896.802	896.811	896.802
Alexandra	727	896.810	896.810	***896*.*800***	896.810	***896*.*829***
Alexandra	1543	896.829	896.829	896.811	896.829	896.811
Alexandra	1969	896.800	896.800	896.815	896.800	896.815
Alexandra	2049	896.815	896.815	896.827	896.815	896.827
Tinkers	13	897.135	897.135	897.173	897.135	897.173
Tinkers	151	897.169	897.169	897.155	897.169	897.155
Tinkers	266	897.163	897.163	897.148	897.163	897.148
Tinkers	547	897.164	897.164	897.148	897.164	897.148
Tinkers	562	897.119	897.119	897.148	897.119	897.148
Tinkers	689	897.173	897.173	897.148	897.173	897.148
Tinkers	923	897.153	897.153	897.144	897.153	897.144
Tinkers	1082	897.101	897.101	897.144	897.101	897.144
Tinkers	1105	897.110	897.110	897.144	897.110	897.144
Tinkers	1283	897.144	897.144	***896*.*423***	897.144	***897*.*141***
Tinkers	1288	897.109	897.109	***896*.*408***	897.109	***897*.*155***
Tinkers	1329	897.164	897.164	897.156	897.164	897.156
Tinkers	1471	897.134	897.134	897.173	897.134	897.173
Tinkers	1548	897.145	897.145	897.163	897.145	897.163
Tinkers	1910	897.141	897.141	897.150	897.141	897.150
Tinkers	2090	896.423	896.423	***897*.*173***	896.423	***897*.*101***

### Parental contribution

**Fig 1:**
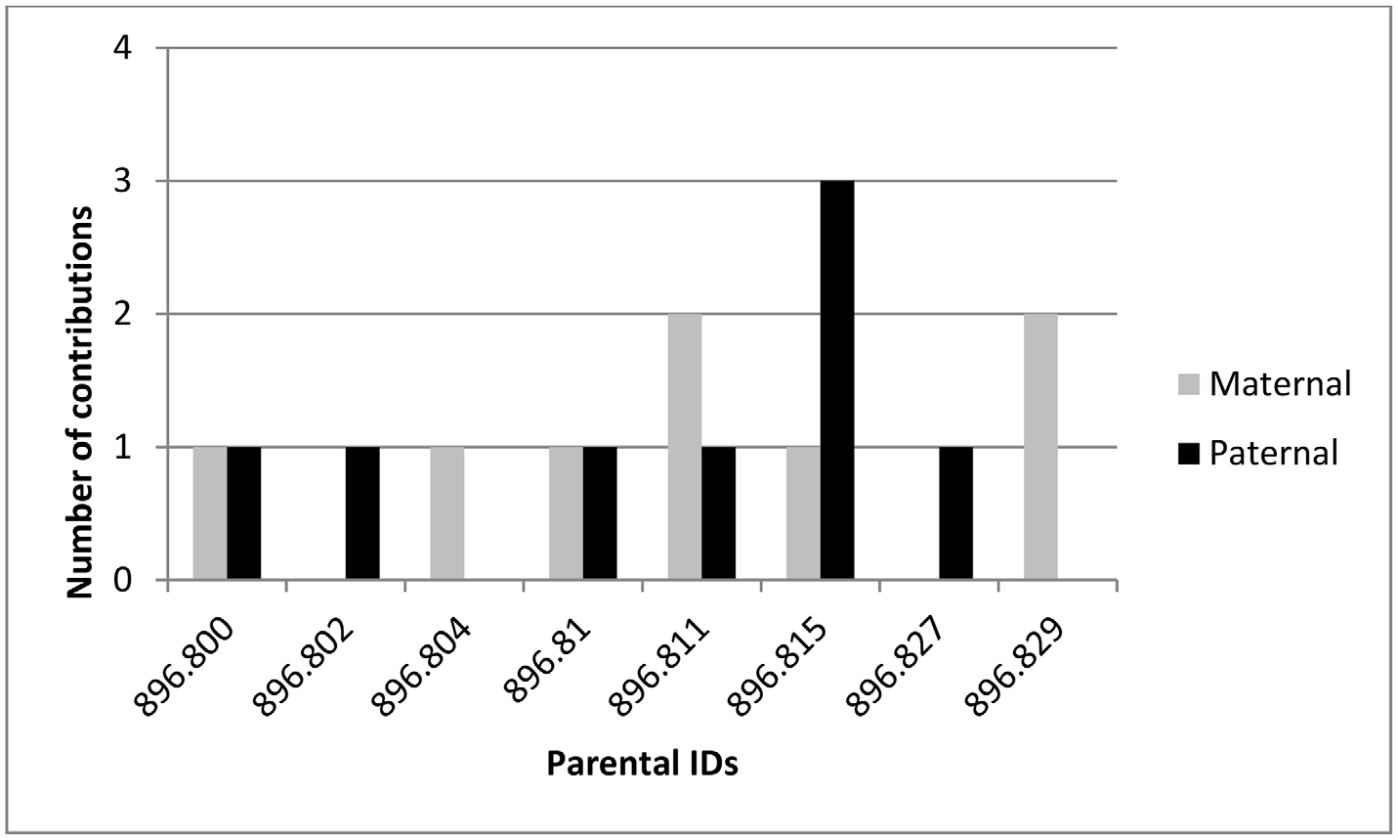
Parental contribution to offspring in the Alexandra seed orchard.

**Fig 2:**
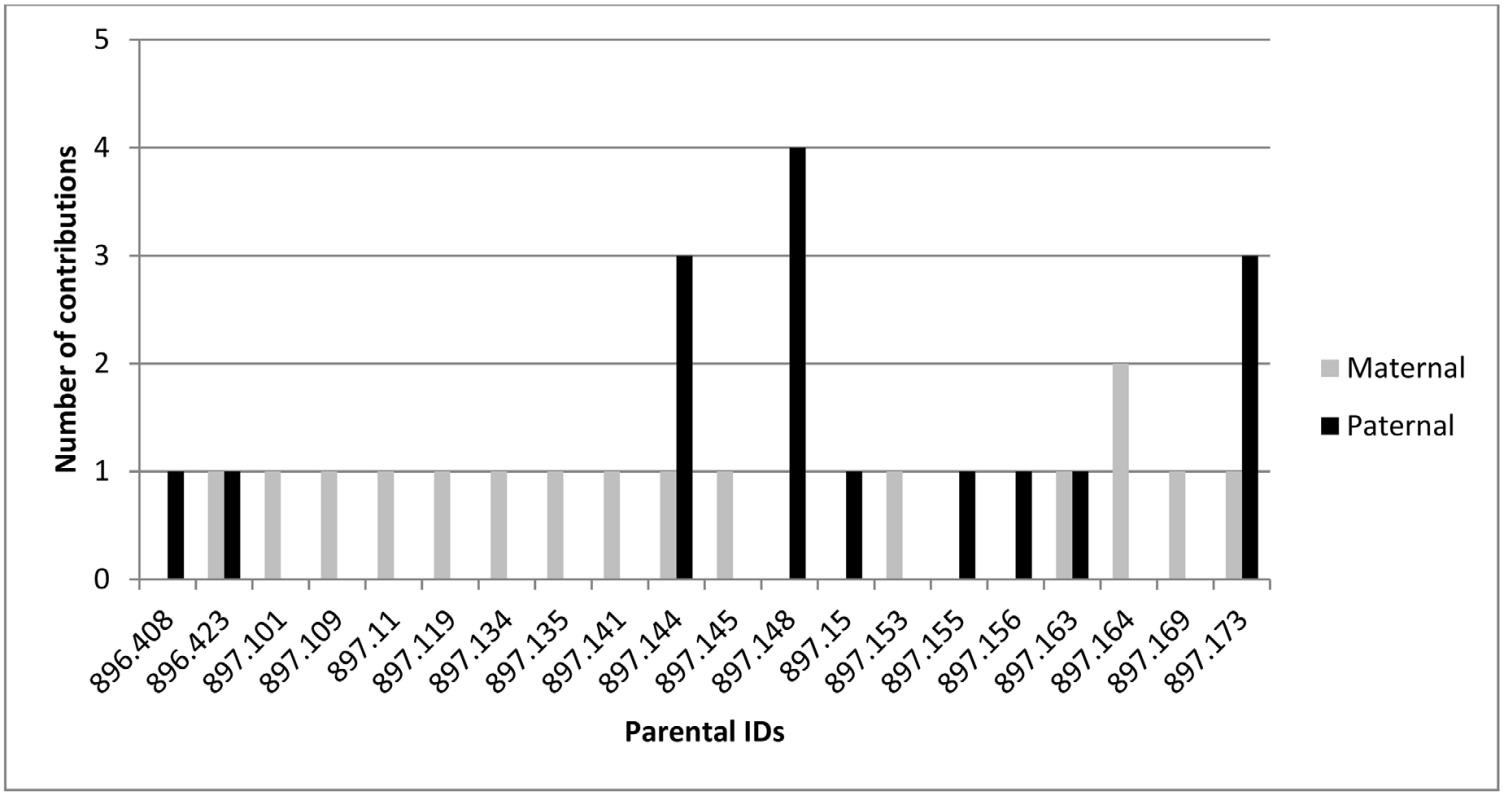
Parental contribution to offspring in the Tinkers seed orchard.

As determined by the SNP markers, the total number of parental contributions from the Alexandra seed orchard, with a marker-supported recorded seed parent is shown in Fig 1. Seed orchard parent 896.815 is the most over-represented (1 maternal contribution and 3 paternal contributions) followed by 896.811 (2 maternal contributions and 1 paternal contribution) and 896.829 (2 maternal contributions). Using the parental assignments made with SNP markers, the total number of parental contributions from Tinkers seed orchard, within selected progeny with marker support for the recorded seed parent, is shown in Fig 2. The most over-represented seed orchard parents are 897.144 and 897.173 (1 maternal contribution and 3 paternal contributions), and 897.148 (4 paternal contributions) followed by 896.423 and 897.163 (1 maternal contribution and 1 paternal contribution).

## Discussion

### SNP vs microsatellite data quality

We generated three different sets of genotypic data for the same set of parents and progenies using the EuCHIP60K [30], the microsatellite kit developed at Scion [1] and a routinely used set of EMBRA microsatellites [36]. These varied in both data quality (call rate, mistyped data) and ability to reconstruct pedigrees, although the ability to determine parentage is clearly dependent on the quality of the data being applied to the reconstruction. In particular, the increase in “missing datapoints” post-2007, which could result from failed amplification reactions or the presence of true null alleles, is a likely contributor to the inability to assign parentage using Scion microsatellite genotypes. Even in individual microsatellite markers, where the number of “missing datapoints” was not visibly high, Hardy-Weinberg analysis indicated higher than expected numbers of homozygotes in some cases. Despite the small number of individuals and their relatedness that could bias HWE expectations, this could imply that genotypes scored as homozygous could be heterozygous for null alleles, and that we were most likely observing true null alleles rather than failed amplifications. The intrinsic dynamics of the PCR amplification efficiency for individual alleles and markers can vary greatly within a single microsatellite multiplex reaction. This variable amplification efficiency in turn may lead to fragment peaks of low signal intensity that do not reach the minimum threshold of relative fluorescence units used to declare an allele call via software such as GeneMapper (Life Technologies). Pre-2007, the genotypes were being scored by eye which provides a degree of subjectivity not available in the automated allele-calling system. This would explain both the increase in putative null alleles and the decrease in heterozygous loci in the post-2007 genotypic dataset particularly at loci where there is differential amplification of alleles. In this particular aspect, large numbers of SNP markers appear to outperform microsatellites with apparent robustness against recording null alleles likely provided by the high redundancy adopted by the Infinium assay where the SNP genotype is actually generated by assembling the signal of several beadtypes across the chip [47]. The clear benefit of microsatellites over SNPs is the amount of information that can be garnered from a single locus, requiring fewer loci to reconstruct a pedigree. However, when fewer loci are genotyped, an undetected null allele has a much greater impact on the exclusionary power of a marker panel. When a sufficient number of marker loci were successfully genotyped (a lower percentage of missing data), the EMBRA microsatellites marker panel was the most successful at assigning parentage confirming the exclusionary power of microsatellite markers reported earlier [25,38]. However the relatively high levels of genotyping inconsistencies due to the intrinsic nature of microsatellite analysis across different laboratories and genotyping platforms, does raise concerns about the reproducibility of microsatellite markers between different operating environments and over time. Furthermore successful multiplexed microsatellite assays are dependent upon the interaction among the multiple primers in the PCR and are sensitive to reaction conditions, reagents and DNA quality. The SNP genotyping assay used in this work, on the other hand, once developed and validated following stringent call rate, inheritance and genotype accuracy parameters [30], is highly optimized, automated and standardized and shows extremely high reproducibility within and across laboratories. Moreover, current SNP assays can be easily transferred across different high-throughput platforms such as from Illumina Infinium to Affymetrix Axiom as recently shown in maize [48]. In this work, genotypes assigned using the Scion microsatellite kit pre-2007 could no longer be used to reconstruct pedigrees of offspring collected and genotyped post-2007 due to the high rate of allele-calling discrepancies (S1 Table). Although some level of allele calling adjustment can be made by using control samples and re-calibration of allele size windows, the robustness of complex microsatellite multiplex assays to changes in software and separation chemistry between and within operators over time is still challenging [10]. Accurate allele calling becomes particularly problematic for dinucleotide repeat microsatellites, subject to microvariant alleles, further complicated in highly polymorphic genomes where SNPs in microsatellite priming sites frequently cause allele dropouts. The development and use of tetranucleotide repeat markers mitigates somewhat this problem, despite the much lower rate of polymorphism [38], but does not solve it completely. This means that the effective ‘shelf life’ of microsatellite genotype data can only be guaranteed with the availability of the same consumables, software and, sometimes, equipment with which they are generated and tends to work better for smaller multiplex panels.

A robust SNP marker genotyping platform, such as the EuCHIP60K, on the other hand provided ample opportunity for choosing extremely robust, informative and independent SNPs. Interestingly, *E*. *nitens* was not one of the main sequenced species wherefrom SNPs were derived for chip development. Nevertheless the high SNP transferability and polymorphism observed across eucalypts of subgenus Symphyomyrtus showed that over 18,000 SNPs were polymorphic in a small sample of 12 trees [30]. Therefore, although a set of 106 SNPs was used in this study, several thousand more SNPs were available with similar performance. These results show that the development of a robust set of SNPs for parentage assignment can be easily made for several other *Eucalyptus* species by simply genotyping with the EuCHIP60K. Equivalent exclusionary power and marker robustness are expected, providing future-proof security for genotypic datasets that are collected over time. Furthermore, the absence of mistyped data in the SNPs data obtained from DNA extracted with different extraction methods, also indicates that the EuCHIP60K is a very robust platform with respect to variation in input DNA quality and generates highly reproducible results.

### Parentage reconstruction

We successfully showed marker support for the recorded seed parent in 8 out of 9 offspring from the Alexandra clonal orchard, and 16 out of 17 offspring from the Tinkers clonal orchard, using a subset of 106 SNP genotypes generated using the EuCHIP60K. In the case of the single unassigned offspring in each of Alexandra and Tinkers orchards, it is highly likely that this arises from a labelling error as there are many places along the chain where these samples may have been mislabelled. The subsequent correct assignment of tree 897.153 as the seed parent of Tinkers progeny 913 indicates this mislabelling occurred in the laboratory, not the nursery or seed orchard. The “chain of identity” for a given sample includes: the parental clone that was placed in the orchard, the progeny that was grown, the samples collected from both putative parent and progeny, and the DNA sample that was extracted and stored. Whilst it may not be practical or possible to identify where a sample was mislabelled, it appears to be happening at a reasonably low level (7%). Mislabelling in other seed orchards ranges from none detected [49], low [50], to 15%- 35% of ramets being mislabelled and planted in the wrong locations [51]. We observed two instances where a recorded seed parent was confirmed, and approximately 10% inconsistent loci were seen between progeny and the most likely pollen parent. It is possible, that pollen from outside the seed orchard was present at a low level in the Alexandra seed orchard. Very low levels (<1%) inconsistent loci were also observed in the most likely trio assignments for three progeny. The single mismatch occurred at the same SNP in all instances, indicating a possible discrepancy with that marker possibly resulting from an ancillary SNP nearby the target SNP that could be impacting the genotype call, although this was not reflected in the GeneCall scores.

### Application of parental information to breeding programme

With the successful identification of male parentage for selected OP offspring, and a desire to limit inbreeding in the next breeding cycle, results show that some parents occur as males up to 4 times (Fig 2), decreasing genetic diversity in the breeding population. To maintain diversity the ‘next best’ tree will be selected using estimated breeding values (Baltunis, unpublished data). These will also be genotyped and the parents determined to ensure diversity levels are maintained in final selections. Using this technology, cost-effective open-pollination can be confidently employed as a breeding strategy, with a diminished risk of inbred forward selections. Ultimately, the aim is to have no one parent contributing more than twice (either as male or female) into the next generation.

The control of inbreeding in *Eucalyptus* is important, as inbreeding levels of up to 36% have been recorded in open-pollinated populations [52]. Ignoring inbreeding will result in inflated additive genetic estimates [53] and a loss of vigour in the population e.g. Chaix et al. [27]. While the use of marker data will now allow the control of inbreeding through limiting the number of related selections in the next generation, we expect that much more will be achievable. Markers can be utilised for adjustments of the relationship matrix and more accurate estimates of genetic variance [53]. SNP sub-sets can be designed for specific genetic studies in *Eucalyptus* including pedigree analysis, linkage and QTL mapping, clone identification, association studies and genomic selection allowing a considerable improvement beyond the current status of genomic research in species of the genus [29].

In conclusion, this study has reported a case study where the power and robustness of microsatellite and SNP genotype data were compared in an operational breeding setting. Besides comparing the performance of the two marker technologies, the data generated were used to inform the management of inbreeding in forward selections of open-pollinated progeny. The 106 SNP markers genotypes used for pedigree reconstruction out-performed the microsatellite marker genotypes for accuracy, reproducibility and robustness. Although specific sets of microsatellites can provide equivalent resolution for pedigree reconstruction, they require considerably more experience and time-consuming analyses to deal with the uncertainties of allele calling, while presenting challenges for data transferability across labs and over time. Still, microsatellite analysis will continue to be useful for breeding applications such as identity analysis and parentage verification due to the high information content, existing infrastructure and low operating costs. The multi-species SNP resource now available with the EuCHIP60k, however, clearly opens a whole new array of opportunities for high-throughput genome-wide or targeted genotyping in species of *Eucalyptus*. With the progressive adoption of genomic data by breeding programs and the continued competition among SNP genotyping platforms, it is expected that SNP genotyping will become increasingly cost-competitive and eventually substitute microsatellite genotyping for most breeding applications.

## Supporting Information

S1 Table Comparison of single sample genotyped with Scion microsatellites in 2007 and in 2011. (DOCX)

S2 Table Comparison of replicate samples genotyped in 2011 with Scion microsatellites. (DOCX)

S3 Table Comparison of replicate samples genotyped in 2013 with EMBRA microsatellites. (DOCX)

S4 Table Comparison of replicate samples genotyped at the EMBRA10 locus at Scion and at EMBRA. (DOCX)
