# Supplementary material for: Parentage Reconstruction in Eucalyptus nitens Using SNPs and Microsatellite Markers: A Comparative Analysis of Marker Data Power and Robustness
Source: PLoS One. 2015 Jul 9;10(7):e0130601. doi: 10.1371/journal.pone.0130601 (PMC4497620; doi:10.1371/journal.pone.0130601)
Supplement: S1 Table — (DOCX) [file pone.0130601.s001.docx]

S1 Table. Comparison of single sample genotyped with Scion microsatellites in 2007 and in 2011

| **MARKER^1^** | **2007** | **2011** |
| --- | --- | --- |
| Eg126a | ***352*** | - |
| Eg126b | ***361*** | - |
| Eg61a | 345 | 345 |
| Eg61b | ***361*** | ***358*** |
| Eg65a | 245 | 245 |
| Eg65b | 245 | 245 |
| Eg98a | ***183*** | ***181*** |
| Eg98b | ***190*** | ***181*** |
| Eg99a | 193 | 193 |
| Eg99b | 193 | 193 |
| EMBRA10a | 118 | 118 |
| EMBRA10b | ***136*** | ***118*** |
| EMBRA39a | ***128*** | - |
| EMBRA39b | ***128*** | - |
| EMBRA64a | 262 | 262 |
| EMBRA64b | 262 | 262 |
| En6a | 94 | 94 |
| En6b | ***99*** | ***100*** |
| Es054a | 102 | 102 |
| Es054b | 104 | 104 |
| ES211a | ***99*** | - |
| ES211b | ***103*** | - |
| FRMSA2a | 115 | 115 |
| FRMSA2b | 115 | 115 |
| FRMSA3a | ***165*** | ***179*** |
| FRMSA3b | 179 | 179 |
| FRMSA4a | ***308*** | - |
| FRMSA4a | ***320*** | - |

1 a and b are alleles of each marker in a diploid individual.

“-” indicate missing data points.
