# Supplementary material for: Parentage Reconstruction in Eucalyptus nitens Using SNPs and Microsatellite Markers: A Comparative Analysis of Marker Data Power and Robustness
Source: PLoS One. 2015 Jul 9;10(7):e0130601. doi: 10.1371/journal.pone.0130601 (PMC4497620; doi:10.1371/journal.pone.0130601)
Supplement: S2 Table — (DOCX) [file pone.0130601.s002.docx]

S2 Table. Comparison of replicate samples genotyped in 2011 with Scion microsatellites.

| **Marker^1^** | **Replicate 1** | **Replicate 2** |
| --- | --- | --- |
| Eg126a | - | - |
| Eg126b | - | - |
| Eg61a | ***345*** | 358 |
| Eg61b | 358 | 358 |
| Eg65a | 245 | 245 |
| Eg65b | 245 | 245 |
| Eg98a | 181 | 181 |
| Eg98b | 181 | 181 |
| Eg99a | 193 | 193 |
| Eg99b | 193 | 193 |
| EMBRA10a | 118 | 118 |
| EMBRA10b | 118 | 118 |
| EMBRA39a | - | - |
| EMBRA39b | - | - |
| EMBRA64a | 262 | 262 |
| EMBRA64b | 262 | 262 |
| En6a | 94 | 94 |
| En6b | 100 | 100 |
| Es054a | 102 | 102 |
| Es054b | 104 | 104 |
| ES211a | - | - |
| ES211b | - | - |
| FRMSA2a | 115 | 115 |
| FRMSA2b | 115 | 115 |
| FRMSA3a | ***179*** | - |
| FRMSA3b | ***179*** | - |
| FRMSA4a | - | - |
| FRMSA4a | - | - |

1 a and b are alleles of each marker in a diploid individual.

“-” indicate missing datapoints.
