# Supplementary material for: Parentage Reconstruction in Eucalyptus nitens Using SNPs and Microsatellite Markers: A Comparative Analysis of Marker Data Power and Robustness
Source: PLoS One. 2015 Jul 9;10(7):e0130601. doi: 10.1371/journal.pone.0130601 (PMC4497620; doi:10.1371/journal.pone.0130601)
Supplement: S3 Table — (DOCX) [file pone.0130601.s003.docx]

S3 Table. Comparison of replicate samples genotyped in 2013 with EMBRA microsatellites.

| **Marker^1^** | **Replicate 1** | **Replicate 2** |
| --- | --- | --- |
| EMBRA2 a | 107 | 107 |
| EMBRA2 b | 107 | 107 |
| EMBRA28 a | 210 | 210 |
| EMBRA28 b | 230 | 230 |
| EMBRA3 a | ***108*** | ***106*** |
| EMBRA3 b | 108 | 108 |
| EMBRA44 a | 182 | 182 |
| EMBRA44 b | 182 | 182 |
| EMBRA11 a | 97 | 97 |
| EMBRA11 b | 127 | 127 |
| EMBRA10 a | - | - |
| EMBRA10 b | - | - |
| EMBRA63 a | 188 | 188 |
| EMBRA63 b | 192 | 192 |
| EMBRA12 a | 127 | 127 |
| EMBRA12 b | 127 | 127 |
| EMBRA157 a | 124 | 124 |
| EMBRA157 b | 124 | 124 |
| EMBRA204 a | 125 | 125 |
| EMBRA204 b | 137 | 137 |
| EMBRA219 a | 265 | 265 |
| EMBRA219 b | 265 | 265 |
| EMBRA4 a | - | - |
| EMBRA4 b | - | - |
| EMBRA128 a | 123 | 123 |
| EMBRA128 b | 129 | 129 |
| EMBRA38 a | 134 | 134 |
| EMBRA38 b | 136 | 136 |
| EMBRA210 a | 194 | 194 |
| EMBRA210 b | 206 | 206 |
| EMBRA681 a | 262 | 262 |
| EMBRA681 b | 268 | 268 |

1 a and b are alleles of each marker in a diploid individual.

“-” indicate missing datapoints
