# Supplementary material for: Parentage Reconstruction in Eucalyptus nitens Using SNPs and Microsatellite Markers: A Comparative Analysis of Marker Data Power and Robustness
Source: PLoS One. 2015 Jul 9;10(7):e0130601. doi: 10.1371/journal.pone.0130601 (PMC4497620; doi:10.1371/journal.pone.0130601)
Supplement: S4 Table — (DOCX) [file pone.0130601.s004.docx]

**S4 Table.** Comparison of replicate samples genotyped at the EMBRA10 locus at Scion and at EMBRA.

| **Sample** | **Scion** | | **EMBRA** | |
| --- | --- | --- | --- | --- |
|  | **EMBRA10a^1^** | **EMBRA10b^1^** | **EMBRA10a^1,2^** | **EMBRA10b^1,2^** |
| 13 | 116 | ***116*** | 116 | ***132*** |
| 91 | 116 | 116 | - | - |
| 151 | 116 | 134 | - | - |
| 166 | 116 | 134 | - | - |
| 262 | 118 | 118 | - | - |
| 266 | - | - | 132 | 136 |
| 326 | 130 | ***131*** | 130 | ***136*** |
| 532 | 134 | 134 | - | - |
| 547 | 134 | 136 | - | - |
| 551 | ***130*** | 130 | ***126*** | 130 |
| 562 | 118 | ***118*** | 118 | ***136*** |
| 572 | 134 | 134 | - | - |
| 689 | 116 | 116 | - | - |
| 727 | 118 | 136 | - | - |
| 913 | 134 | 134 | 134 | 134 |
| 923 | 130 | 134 | - | - |
| 1074 | 130 | 130 | - | - |
| 1082 | 130 | 130 | - | - |
| 1105 | 134 | 134 | - | - |
| 1178 | 118 | 136 | - | - |
| 1283 | ***130*** | ***130*** | ***134*** | ***134*** |
| 1288 | 118 | 134 | 118 | 134 |
| 1329 | - | - | - | - |
| 1446 | 134 | 134 | - | - |
| 1471 | 116 | 116 | - | - |
| 1543 | - | - | - | - |
| 1548 | 130 | 130 | - | - |
| 1742 | 130 | 134 | - | - |
| 1870 | 116 | 116 | - | - |
| 1910 | - | - | 132 | 136 |
| 1950 | 134 | 136 | - | - |
| 1963 | 130 | 134 | 130 | 134 |
| 1969 | 116 | 118 | - | - |
| 1978 | 134 | 135 | - | - |
| 2049 | 116 | 134 | - | - |
| 2090 | 118 | 134 | - | - |
| 896.408 | 134 | 134 | - | - |
| 896.423 | 134 | 134 | - | - |
| 896.800 | 118 | 134 | - | - |
| 896.802 | 130 | 134 | 130 | 134 |
| 896.803 | 134 | 134 | - | - |
| 896.804 | ***116*** | 130 | ***118*** | 130 |
| 896.806 | 134 | ***136*** | 134 | ***138*** |
| 896.806 | 130 | 130 | 130 | 130 |
| 896.807 | - | - | - | - |
| 896.810 | 118 | 118 | - | - |
| 896.811 | 134 | 134 | - | - |
| 896.815 | - | - | - | - |
| 896.816 | 130 | 130 | 130 | 130 |
| 896.821 | - | - | 132 | 132 |
| 896.822 | 134 | 134 | - | - |
| 896.826 | 130 | 130 | 130 | 142 |
| 896.827 | 134 | 134 | - | - |
| 896.828 | 130 | 130 | - | - |
| 896.829 | 118 | 118 | - | - |
| 896.829 | 118 | 118 | - | - |
| 897.101 | 130 | 134 | 130 | 134 |
| 897.109 | 116 | 118 | 116 | 118 |
| 897.110 | 130 | ***130*** | 130 | ***132*** |
| 897.113 | 130 | 134 | - | - |
| 897.119 | 118 | 118 | - | - |
| 897.124 | - | - | 136 | 136 |
| 897.129 | ***134*** | 134 | ***132*** | 134 |
| 897.134 | 116 | 116 | 116 | 116 |
| 897.135 | 116 | ***116*** | 116 | ***136*** |
| 897.141 | 134 | 134 | - | - |
| 897.142 | 134 | ***134*** | 134 | ***136*** |
| 897.143 | 130 | 130 | - | - |
| 897.144 | 130 | 134 | - | - |
| 897.145 | 130 | 130 | 130 | 130 |
| 897.148 | 134 | 134 | - | - |
| 897.150 | 118 | 132 | - | - |
| 897.153 | 134 | 134 | - | - |
| 897.155 | 116 | ***116*** | 116 | ***136*** |
| 897.156 | 130 | 130 | - | - |
| 897.158 | ***134*** | 134 | ***132*** | 134 |
| 897.161 | 130 | 130 | - | - |
| 897.163 | 134 | 134 | - | - |
| 897.164 | 136 | 136 | - | - |
| 897.168 | 116 | 130 | - | - |
| 897.169 | 134 | 134 | 134 | 134 |
| 897.173 | 116 | ***116*** | 116 | ***132*** |
| 897.174 | 134 | 134 | - | - |
| 897.177 | 130 | 134 | - | - |

1 a and b are alleles of each marker in a diploid individual. “-” indicate missing

datapoints

2 EMBRA allele calls adjusted by 7bp to match the 7bp longer reverse primer

used at Scion
